# Survey and Diagnostic Challenges after Transmission-Stop: Confirming Elimination of *Schistosomiasis haematobium* in Morocco

**DOI:** 10.1155/2020/9705358

**Published:** 2020-01-22

**Authors:** Amarir Fatima, Balahbib Abdelaali, Paul L. A. M. Corstjens, Sadak Abderrahim, Adlaoui El Bachir, Rhajaoui Mohamed

**Affiliations:** ^1^Department of Parasitology, National Reference Laboratory for Schistosomiasis and Malacology, National Institute of Hygiene, Agdal, Rabat, Morocco; ^2^Department of Biology, Laboratory of Immunology and Biodiversity, Faculty of Sciences Ain Chock, University Hassan II, Casablanca, Morocco; ^3^Laboratory of Zoology and General Biology, Faculty of Sciences, Mohammed V University in Rabat, Morocco; ^4^Department of Cell and Chemical Biology, Leiden University Medical Center, Leiden, Netherlands

## Abstract

Clinical cases of Moroccan residents have been recorded since 2004, indicating successful interruption of transmission of *S. haematobium* infection at national level. The first national survey initiated in 2009 for* Schistosomiasis haematobium* among children born after 2004, applied diagnostic test was the HAMA-EITB, based on the Western blot technology, and molecular malacological diagnostic tools clearly confirm transmission stop. In 2015, a recent, small survey utilizing an HAI, ELISA tests and an ultrasensitive antigen test, FTCUP CAA, in a group of individual with a past history of infection. However, obviously follow-up surveys to prevent reemergency and for certification of the schistosomiasis elimination require vigilant diagnosis strategies. Here we discuss diagnosis story line in the national laboratory and challenges based on the available tools in relation to their clinical parameters (sensitivity/specificity; Sn/Sp), practicability and associated costs. When transmission stop has been achieved, survey cost and speed are likely to benefit from cost effective pooling strategies and ultrasensitive assays indicating active infection in all potential risk groups. Similarly molecular pooling strategies to monitor infections in the snail vectors.

## 1. Introduction

*Schistosomiasis haematobium* is a worldwide public health problem affecting 150 million persons in third-world countries. It was estimated that annual mortality due to nonfunctioning kidneys caused by infections with *S. haematobium *could be as high as 150,000 [1, 2]. Although morbidity and mortality was significantly decreased after introduction of programs that include large-scale drug therapy, estimates are that in 2015 at least 218 million people still require preventive treatment to restrain transmission [3]. National programs applying repetitive (annually or bi-annually) mass drug administration (MDA) approaches have been implemented in several countries in Africa [4]. The immediate impact of (repeated) chemot.herapy in case of *S. haematobium *infection is a decrease in the number of severe cases of urinary schistosomiasis and the concurrent reduction of the parasite load, in general this leads to overall lower endemicity. However, long term success of any MDA approach depends on additional measures as behavioral changes, improved sanitation and access to clean fresh water. As schistosomiasis is a vector-borne disease, actions to constraint or eliminate the intermediate host (i.e., snails from the genus *Bulinus*) are apparently mandatory as well. In MDA low endimicity established settings, new- and re-infections will likely continue when the secondary measures are not sufficient. Moreover, new cases and low worm burden may not be recognized effectively because they are not easily detected by the generally used low sensitive egg microscopy and poor specificity of serological (antibody detection) methods. In these settings the proper timing of MDA-stopping decision is difficult when evidently the required appropriate diagnostic tools for monitoring are missing. Highly sensitive diagnostic tools are therefore required to successfully proceed-to and validate the elimination of the disease [5].

Nevertheless, in several countries, morbidity has been successfully controlled and transmission thought to be interrupted as a consequence of intensified efforts. The status of schistosomiasis in areas that have reached low-transmission was reviewed not long ago [6] and recommendations offered regarding tools and strategies for monitoring, criteria to determine interruption of transmission and validation of elimination. However, these recommendations do not (yet) include the implementation of the newly developed ultrasensitive diagnostic tools [7, 8] that allow highly accurate determination of the infection status [9–15]. It is believed that transmission- stop has been achieved in 19 countries, and currently some already await confirmation of successful steep towards elimination. An unequivocal approach including the use of accepted diagnostic tools is crucial to demonstrate actual elimination status. The aim of our review is to present the line story of the disease in Morocco, the description of the diagnostic considerations for verification of elimination, and cost effective diagnosis strategy purposed to certify schistosmiasis elimination.

## 2. Schistosomiasis Transmission Interruption in Morocco

Morocco is one of the countries thought to progress rapidly towards elimination. Urinary schistosomiasis caused by *S. haematobium *and transmitted by *Bulinus truncatus*, was first diagnosed in 1914 in Marrakech. It became highly endemic and was given priority in public health work in the early eighties [16]. The intervention was successful, and at 2004, the interruption of transmission at national level was apparently achieved. Since 2004 no new *Schistosoma *infection in Morocco of a native resident has been recorded.

Five years later, an initiative to confirm interruption of transmission and first step to towards endorsement of elimination was started in 2009 with a national serological survey for *Schistosomiasis haematobium* among children born after 2004. The areas selected to participate were the past schistosomiasis endemic provinces: Errachidia, Tata, El Kelaa des Sraghna, Beni Mellal, and ChtoukaAit Baha. The applied diagnostic test was the HAMA- EITB, based on Western blot technology. The test screens for the presence of antibodies against microsomals *S. haematobium *worm antigens with presumptive clinical sensitivity and specificity of 99%. The results demonstrated absence of new antibody positive cases for all 2382 individuals included in the survey [17]. A sometime later performed national malacological survey that simultaneous use of DraI PCR and the specific Sh110/Sm-SL nucleic acid PCR test in past endemics areas in Morocco indicated absence of *S. haematobium *in its specific snail vector *Bulinus truncatus*. Although the survey indicated the potential presence of *S. bovis*, results are considered good confirmation for transmission stop of human infections by *S. haematobium *as also indicated by the serological survey [18]. Hence, these two surveys and the fact that no new cases of urinary schistosomiasis have been reported suggested successful elimination of the disease in Morocco. Then obviously follow-up routine surveys to prevent reemergency require vigilant and cost effective diagnosis strategies.

## 3. Post Elimination (Diagnostic Tests Challenges)

### 3.1. Antibody Detection Test

Antibody detection as recommended by WHO [19] obviously is well suited to test individuals born after transmission stop followed by antigen or nucleic acid based testing for those testing seropositive cases [20]. However, antibody testing for travelers, immigrants and residents born before transmission stop was claimed does not seem to be a valid approach as there are no accurate antibody tests that can distinguish past from previous infection. Moreover, sensitivity as well as specificity of the current antibody assays may be an issue [21, 13]. Relevantly large inconsistencies between results obtained with different antibody tests still make the general application of a single antibody test challenging [22]. Algorithms based on the use of multiple antibody tests would be needed to obtain the required high sensitivity. When targeting maintaining transmission stop, specificity seems somewhat less important as treatment of few false positives is not disastrous although surely not recommendable in view of possible drug side effects and potential drug resistance. However, to certify elimination specificity cannot be neglected. Appropriate algorithms based on parallel rather than serial testing may partly deal with specificity issues but it will relevantly increase cost. HAMA EITB is highly sensible and specific, using pooling samples, but strep test preparation required worm *Schistosoma haematobium* culture and high technology (the antigen must be ordered from committed international laboratories one year before). Antigen and to a lesser extend nucleic acid based testing may then become valid alternatives especially when high sensitivity and specificity can be warranted even when testing using pooling strategies. As is the case in several other countries, in Morocco the tool used for routine monitoring is still the direct examination of the urine of children with a focus on the (previous endemic) high risk areas. The detection of the presence of viable eggs in urine defines an active case [19]. Given the large migration of African refugees from endemic countries during the last decade and the real probability to miss the low-worm/egg burden cases, it is prudent to improve the epidemiological tools for children serological survey in high risk areas.

### 3.2. Antigen Detection Test

In 2017, small study amongst 37 adults with a history of past infection (before 2004) indicated 2 individuals still carrying residual but low worm burden *Schistosoma *infections; the actual involved species was not determined [13]. The applied diagnostic tool detected a worm-specific carbohydrate which is discharged by alive worms into the human blood circulation, the circulating anodic antigen (CAA). This bio-molecule is detectable in blood and/or urine and indicates an active ongoing infection, but does not always imply egg production. The CAA test is a highly specific and the most sensitive assay capable to detect a single worm *Schistosoma *infection including non-fecund or single-sex worms. It is a genus specific test that will identify active infections with any of the know *Schistosoma *species, including *bovis*, *curassoni *and *intercalatum *[13]. The identification of the two individuals with residual infection indicates the importance of the need of proper and highly sensitive diagnostic tools to obtain accurate information regarding elimination status. The infected individuals were non-symptomatic and likely carry a low worm burden as indicated by the measured level of CAA, with worms probably incapable of egg production. However, it seems clear that to maintain the currently alleged elimination of urogential schistosomiasis in Morocco and to prevent reemergency of infection either through unexpected species, residual low worm burden infections in adult residents, or through infected immigrants and travelers, a highly focused strategy and survey protocol is needed.

## 4. Selecting Suitable Diagnotic Tools for Current Surveys

Surveys may include parasitological methods, serology (antibody detection) and any newly available test as e.g. assays detecting worm antigen in bodily fluids. The surveys should also include snail surveys as presence of the vector by itself does provide an important pulpit for re-emergence of the disease. Besides Sn and Sp issues of the applied tests, costs issues play a role and in part determine the scale at which the surveys can be performed. Possibilities to implement accurate and cost-effective pooling strategies may thus be of importance. For the surveys three types of assays are considered: (i) assays detecting active *Schistosoma *infection for individuals born before 2004 and immigrants; (ii) assays detecting exposure to *S. haematobium (Sh)* and *S. bovis (Sb)* for individuals born after 2004; (iii) assays detecting snails infected with miracidia. In Table 1 a number of tests are listed that were previously used, and/or are considered for future use in national surveys to detect *Schistosoma *infection. We note that not all tests are (always) commercially available and price-ranges can be quite variable, but obviously when surveys are part of scientific studies (including the evaluation of laboratory based ‘research-only' tools) or *vice versa*, noncommercial diagnostics should not be excluded.  Table 2 summarizes potential tests available for malacological surveys to detect infected snails.

- Assays detecting active* Schistosoma* infection for individuals born before 2004 and immigrants: For post-transmission and elimination settings, it is recognized that parasitological test (urine microscopy in case of Morocco) does not provide good enough sensitivity and cannot be considered an accurate tool. Detection of circulating antigens, i.e. the circulating anodic antigen detected with the UCP-LF CAA test platform, has demonstrated excellent performance worldwide in different endemic and no endemic settings. CAA detection assay formats are available for dried blood samples [42], serum, plasma and urine [11–13]. The test includes a sample preparation step which may make the assay in its current format somewhat less field-friendly as this step requires some basic laboratory equipment. However, the sample preparation step makes the assay highly flexible towards sample input (matrix type and volume) allowing improved sensitivity to the level that single-worm, single -sex and low fecundity infections are detectable. This flexibility towards sample amount also makes the assay well suited for cost-effective pooling strategies [43].
- Assays detecting exposure to* S. haematobium* and* S. bovis* for individuals born after 2004*: *In China where *S. japonicum (Sj) *has been largely eliminated as a public health problem in through implementation of a series of different measures [44]. Detection of seropositive individuals is applied to identify potential active infections and transmission. However, a study by van Dam et al. indicated that the applied antibody test lacks specificity [21]. HAMA EITB highly specific and sensible but could be expensive. A generic rapid antibody detection platform utilizing UCP-LF has been developed for testing with various biological matrices (blood-based, urine and saliva) [7–46], but still require evaluation and validation for potential application in pooling approaches. The antibody assays do not need a sample preparation step and are usable for point-of-care (near patient) use and if needed can be performed non-invasively using urine samples [47].
- Assays detecting snails infected with miracidia.

Malacological studies utilizing nucleic acid (NA) amplification to detect miracidia NA are also well suited for pooling strategies as the technology also requires a sample purification step which is quite flexible towards sample volume. Simultaneous use of DraI PCR, and Sh110 SmSl PCR, as used in our first study, take more time and reactive [18]. More recently, Abassi et al. develop a simple and more sensitive PCR assay that enables direct discrimination of *S. haematobium *from related animal schistosomes, by the primer combination of DraI reverse primer and Sh73 direct primer (73d). The sensitivity of *S. haematobium*detection was 1 pg/*μ*l, whereas *S. bovis *detection was 10 pg. It can be assumed that detection of snails infected with *S. haematobium *will be accomplished from very early prepotency. Such assays required further validation using larger numbers of field snails in Morocco [41, 18].

## 5. Survey to Certify Elimination

### 5.1. Human Survey

**Figure 1:**
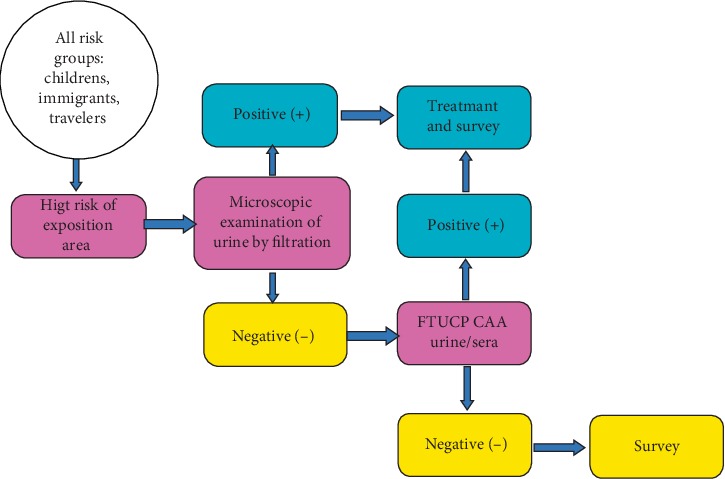
Approach of parasite survey in post elimination phase of *Schistosomiasis haematobium* in Morocco.

The process of confirming the interruption of transmission and possible certification of elimination is based on human and snails survey each five years since 2009, with the third survey coming up in 2019. The national survey of *Schistosomiasis haematobium *is largely focused on children born after transmission-stop 2004. Surveys will be performed in settings in past endemics provinces and in areas were imported African cases were registered during the last years. As diagnostic test, besides individual urine parasitological, the UCP-LF antigen assay for CAA detection also in individual sera and urines (Figure 1).

### 5.2. Malacological Survey

Belkacemi et al. reported that March is the month of reproduction of snails in Morocco, and juvenile snails become infective in the beginning of September [48, 49].Transmission period is greatly associated with population movement generally important in summer August-Octobre (holiday, travel, swim, irrigation) [49, 50]. Survey will be focused on *Bulinus truncatus*, the intermediate host of schistosomiasis in Morocco, and *Planorbarius metidjensis*, experimental intermediate host of *S. haematobium* in south of Morocco.

PCR of snail pools has been shown to be practical for large-scale monitoring of *S. haematobium* transmission, however there is a need to improve the cost-effectiveness of the test by development of a method similar to LAMP amplification that is cheaper and more field friendly [40]. The malacological treatment may be done annually in each breeding sites showing *Bulinus* in the last three years, because, treatment can left the juvenile snails, and the life age of *Bulinus* is estimated to two years [51, 52].

## 6. Conclusion

Schistosomiasis antigen detection test and malacological molecular test are suitable for human and snail survey in post elimination phases. Nevertheless to consolidate the approach survey, temporary drying of irrigation canals, sanitary education, improvement of Schistosomiasis laboratories, improvement of basic formation and training programs (hours number) of medical parasitology for students in institute of technical health even if some parasitic disease are eliminated, improvement of scientific technology, survey of imported cases and monitoring of risk map may be useful to maintain elimination, prevent reemergence of the disease and for certification of the schistosomiasis elimination.

## Figures and Tables

**Table 1:** Considered diagnostic tests for *Schistosomiasis haematobium * in the Moroccan survey.

Diagnostic	Test	Field	Cost/test (USD)	Evaluation	Sensitivity/specificity	Note	Ref
Clinical test	Hem test^1^	**+**	**0, 3**	Iraq, Ghana, Sudan, Tanzania, Nigeria, Sub Saharan Africa	79%/98%	Morbidity test	[26]

Antibodies detection	HAMA EITB^2^	_	>5	Morocco USA	95%/100%	High cost and not field-friendly	[12, 23]
	EITB/WB 23kDa^3^	_	8, 8	Egypt and Morocco	50/91%	High cost and not field-friendly	[23, 24]
	ELISA-SEA^4^ (soluble eggs antigen)	**+**	8, 8	Ghana	98.5%/83%/93%	High cost	[23, 25]
						Need laboratory	
	HAI^5^(whole adult worm antigen)	**+**	2.5	Germany, Chile and Netherlands	92%/94.7%	Acceptable	[25–26]
	DDIA^6^	**+**	1	China	--	Need evaluation in *haematobium* low endemic areas	[28]
	RDT SmCTF^7^	**+**	**<3**	Ivory Coast	66.7%/34.4%	Not acceptable	[29]

Antigen detection	Filtration	_	**<3**	Africa, Iran, Iraq		Need laboratory	[30]
	RDT filtration	**+**	**<3**	USA, Kenya	79%/95%	Field friendly and cost effectiveness, but not hygienic in large number of samples	[31]
	POC CCA^8^	**+**	**3**	Cameroun, Ivory Coast Ethiopia, Kenya, Uganda	36%/78%	Not sensitive in *haematobium* low endemic areas	[32, 33]
				Zanzibar	99%		[30]
	PCR^9^	_	8	Brazil	100%	High cost	[20, 33]

1. Urine hemedipstick, 2. Heamatobium microsomal antigen enzyme Immunotransfert Blot, 3. 23 kDa Western blot, 4. Soluble eggs antigen enzyme linked

immunosorbent assay, 5. Hemmaglutination indirect, 6. Dipstick dye immunoassay, 7. *Schistosoma mansoni* cercarial transformation fluid, 8. Point of care/circulating cathodic Antigen, 9. Polymerase chain reaction.

**Table 2:** Considered tests for malacological diagnosis of *S. haematobium *infection.

Test	Prepatent infection detection	Sensitivity	Discrimination between *S. haematobium *and *S. bovis*	Field friendly (large survey)	Cost USD	References
Light test	No	1 Miracidium	No	No	0	[33]
Dissection	Yes	1 Sporocyste	No	No	0	[34]
Hemolymph test	Yes (after 2 weeks)	Seropositivite	No	No	5	[35–37]
DraI PCR	Yes	1 pg/*µ*l	No	Yes	7	[38]
Sh110SmSl PCR	Yes	1 pg/*µ*l	Yes but *S. bovis not amplified*	No	7	[39]
DraI et Sh110SmSl PCR	Yes	1 pg/*µ*l	Yes (amplify *S. bovis*)	Yes	14	[18]
PCR 77/73	Yes	1pg/*µ*l	Yes (amplify *S. bovis*)	Yes	7	[40]
CoxI PCR		0.8ng/*µ*l			8	[2]
